# Maternal Supraphysiological Hypercholesterolemia Is Accompanied by Shifts in the Composition and Anti-Atherogenic Functions of Maternal HDL along with Maternal Cardiovascular Risk Markers at Term of Pregnancy

**DOI:** 10.3390/antiox12101804

**Published:** 2023-09-27

**Authors:** Claudette Cantin, Andrea Morales, Ramón Serra, Sebastián E. Illanes, Andrea Leiva

**Affiliations:** 1 School of Medical Technology, Faculty of Medicine and Science, Universidad San Sebastián, Santiago 7500000, Chile; 2 Hospital Naval, Punta Arenas 6200000, Chile; 3 Faculty of Medicine, Universidad de los Andes, Santiago 111711, Chile; sillanes@uandes.cl; 4 Laboratory of Reproductive Biology, Center for Biomedical Research and Innovation (CIIB), Universidad de los Andes, Santiago 111711, Chile; 5 IMPACT, Center of Interventional Medicine for Precision and Advanced Cellular Therapy, Santiago 8331150, Chile

**Keywords:** pregnancy, high-density lipoproteins, atherogenic profile, vascular dysfunction, cardiovascular risk

## Abstract

Background: Maternal physiological hypercholesterolemia (MPH) occurs in pregnancy for a proper fetal development. When cholesterol increases over the physiological range, maternal supraphysiological hypercholesterolemia (MSPH) is described, a condition underdiagnosed by a lack of evidence showing its biological and clinical relevance. Aim: To determine if MSPH associates with maternal vascular dysfunction, along with changes in the composition and function of maternal HDL leading to increased cardiovascular risk. Methods: This study included 57 women at term of pregnancy in which a lipid profile was determined. Results: Maternal total cholesterol (TC) and LDL but not HDL were increased in MSPH women. The isolated HDL from a subgroup of MSPH women had a lower protein abundance and a reduced activity of the antioxidant enzyme PON1; however, an increased antioxidant capacity compared to MPH was observed, along with higher serum levels of α-tocopherol. Moreover, HDL from a subgroup of MSPH women had a lower capacity to induce NO synthesis in endothelial cells compared to MPH. In the circulation, we observed a reduced total antioxidant capacity and augmented levels of soluble VCAM, ApoB, ApoCII, ApoCIII, IL-10, and IL-12p70, as well as the cardiovascular risk ratio ApoB/ApoAI, compared to MPH women. Conclusion: MSPH women present dysfunctional HDL and increased atherogenic cardiovascular risk factors.

## 1. Introduction

During pregnancy, maternal cholesterol levels increase physiologically due to fetal cholesterol requirements, a condition referred to as maternal physiological hypercholesterolemia (MPH, maternal total cholesterol (TC) at term ≤280 mg/dL) [1,2,3]. However, in some pregnancies and by mechanisms that are under study, maternal TC levels increase over the physiological levels, a condition regarded as maternal supraphysiological hypercholesterolemia (MSPH, TC at term >280 mg/dL) [4,5,6], which is mainly associated with increased low-density lipoprotein (LDL) cholesterol levels. This cut-off point has been established mainly based on the negative consequences of MSPH on the fetoplacental vasculature: endothelial dysfunction of both the macro- and microvascular placental vessels [6,7], oxidative stress in neonatal blood and placental tissue [8,9], impaired cholesterol traffic through placental trophoblasts [10]. Regarding atherosclerotic cardiovascular disease, MSPH associates with larger atherosclerotic lesions in the arteries of fetuses and children of these women, together with an increased severity of myocardial infraction in adulthood compared to MPH offspring [1,11,12]. Even though there is wide evidence regarding the consequences of the MSPH condition on fetoplacental vasculature, little is known about its effects on the vascular health of these women, including atherosclerotic cardiovascular disease. In this regard, a recent study reported elevated levels of lipid peroxidation in the serum of MSPH women along with a pro-atherogenic profile of maternal LDL, which contribute to a pro-oxidative environment compared to MPH [13]. However, it is unknown whether the MSPH condition at term of pregnancy is related with maternal endothelial dysfunction and inflammatory markers, conditions associated with the genesis of atherosclerotic cardiovascular disease.

In adults, high-density lipoproteins (HDLs) have been extensively studied as important factors that can prevent atherosclerosis and cardiovascular disease (CVD) progression, not only because HDL cholesterol levels have been inversely associated with both CVD and mortality, in a U-shaped manner [14], but also since HDLs exert multiple anti-atherogenic functions on cells important to the atherosclerotic process, such as endothelial cells [15]. During pregnancy, maternal HDL cholesterol levels remain relatively stable, the predominant HDLs being HDL type 2, which are enriched in triglycerides (Tgs) and have a large size compared to the smaller and denser HDL type 3 that are more abundant in non-pregnant women [16,17,18]. In addition, recent data has shown that pregnancy is not only associated with changes in the size of HDL but also with dramatic shifts in diverse protein clusters across different HDL subfractions compared to non-pregnant controls [18]. Regarding HDL from MPH and MSPH women, previous reports showed that HDL cholesterol levels are similar between both groups of women [10,13,19]. However, if MSPH associates with changes in the composition or function of maternal HDL is unknown, changes at this level could have a negative impact on endothelial cells and on the vascular health of these pregnant women. The above is particularly important since these compositional changes could have major implications on HDL anti-atherogenic functions, such as cholesterol efflux capacity, antioxidant capacity, anti-inflammatory activity, and vasodilatory activity [20,21].

Thus, the aim of this work was to determine whether MSPH is associated with maternal vascular dysfunction at term, along with changes in the composition and function of maternal HDL leading to increased cardiovascular risk.

## 2. Methods

### 2.1. Study Groups

Human samples (maternal blood) were obtained from 57 pregnant women from Clínica Dávila, Chile, at term of pregnancy. For the different determinations in plasma were used 15 samples per group, and for HDL in vitro assays were used 8 samples per group. The sample size calculation was performed by using the mean difference criteria and considering that MSPH has been reported in a 25% of pregnant women. Therefore, 60 women were recruited to have at least 15 MSPH women.

The investigation conformed to the ethical guidelines of the 1975 Declaration of Helsinki. Ethics approval was obtained from the Faculty of Medicine of the Pontificia Universidad Católica de Chile (PUC; ID 190426004) and Clínica Dávila (ID 180810004), and informed consent from patients was obtained. General maternal (i.e., age, height, weight, blood pressure, and glucose levels) and neonatal (i.e., sex, gestational age, weight, and height) variables were obtained from the clinical records by the medical staff.

Pregnant women with TC < 280 mg/dL at term of pregnancy were included in the MPH group (*n* = 34), and those with TC ≥ 280 md/dL in the MSPH group (*n* = 23). The cut-off value for MSPH reflects the values at which human fetoplacental endothelial and vascular dysfunction have been previously reported [1,2,5,6,8,9,10,11,13]. The exclusion criteria were maternal obesity at term of pregnancy, excessive weight gain between the first and third trimester of pregnancy, pregestational and gestational diabetes (GDM), preeclampsia, intrauterine growth restriction, fetal malformations, and other maternal pathologies, as described previously [13].

### 2.2. Determination of Maternal Cholesterol and Triglyceride Levels

TC, HDL, LDL, and triglyceride levels were determined in maternal blood obtained from brachial venous blood, as described [7]. Lipid determination was performed in the Clinical Laboratory of the HCUC via standard enzymatic colorimetric assays, as previously described [13].

### 2.3. HDL Isolation

HDL from MPH and MSPH women was isolated by ultracentrifugation, as described [13]. Briefly, sucrose (final concentration: 10%), EDTA (10 mmol/L, pH 7.4), aprotinin (2 μg/mL), and phenylmethylsulfonyl fluoride (PMSF, 1 mmol/L) were added. The serum density was adjusted to 1.24 g/mL with potassium bromide (KBr). A gradient was generated by the addition of PBS (2.6 mL, density: 1.006 g/mL) to the serum (1.4 mL, density: 1.24 g/mL). The gradients were ultracentrifugated at 281,000× *g* RCF at 15 °C for 4 h (TH-660 Thermo-Sorvall rotor). After centrifugation, the corresponding bands of LDL and HDL were collected from the gradients. Isolated lipoproteins were dialyzed in saline solution (mmol/L 150 NaCl, 0.34 EDTA, pH 7.4, 4 °C, 48 h), and stored at 4 °C in sealed tubes saturated with nitrogen. The protein concentration was determined as described in the Western blot section. The correct isolation of HDL was determined by SDS-PAGE separation followed by Western blot for ApoB and ApoAI.

### 2.4. Human Umbilical Vein Endothelial Cell Culture

Human umbilical vein endothelial cells (HUVECs) were isolated by collagenase II digestion from umbilical cords obtained from normal-term pregnancies at birth, as described [8]. The cells were cultured (37 °C, 5% CO_2_) in medium 199 (M199; Gibco, New York, NY, USA), containing 5 mmol/L D-glucose, 10% newborn calf serum (NBCS), 10% fetal calf serum (FCS) (Gibco, USA), 3.2 mmol/L L-glutamine, and 100 U/mL penicillin-streptomycin (primary culture medium, PCM) (Gibco, USA). Twenty-four hours prior to experiments, the incubation medium was changed to 5% NBCS/FCS containing M199.

### 2.5. Endothelial Cell Line Culture

Human dermal microvascular endothelial cell 1 (HMEC-1) was cultured (37 °C, 5% CO_2_) in MCDB131 medium (Sigma-Aldrich, St. Louis, MI, USA), supplemented with hydrocortisone (1 μg/mL; Sigma-Aldrich, St. Louis, MI, USA), recombinant human endothelial growth factor (EGF; 1 ng/mL; Gibco, USA), fetal bovine serum (FBS; 10% (*v*/*v*), Gibco, USA), and penicillin-streptomycin (100 U/mL) (Lubrano et al., 2012), as described [19]. Experiments were performed in a medium without FBS (minimum medium).

### 2.6. Protein Quantification and Western Blot

Protein abundance was determined in isolated HDL and in homogenized cells, as described [19]. Confluent cells were lysed with KOH (0.5 N) or in RIPA protein lysis buffer (Sigma-Aldrich, St. Louis, MI, USA), adding protease and phosphatase inhibitors (Sigma-Aldrich, St. Louis, MI, USA). Cells were sonicated (6 cycles, 5 s, 100 W, 4 °C), and total protein was separated by centrifugation (14,000× *g*, 10 min, 4 °C). Protein content was determined with Bradford reagent (Bio-Rad, Hercules, CA, USA) or a bicinchoninic acid (micro BCA) protein assay kit (Thermo Fisher Scientific, Waltham, MA, USA).

The proteins (30 μg) were separated by polyacrylamide gel electrophoresis (8% and 20%) in denaturing and reducing conditions. Subsequently, the proteins were transferred to polyvinylidene difluoride membranes and later probed with primary rabbit polyclonal anti-apolipoprotein B (ApoB; 1:1000 dilution), anti-apolipoprotein AI (ApoAI; 1:1000 dilution), anti-apolipoprotein AII (ApoAII; 1:1000 dilution, loading control) (Abcam, Cambridge, UK), anti-total eNOS (1:500 dilution), anti-eNOS phosphorylated at Serine^1177^ (p-Ser^1177^; 1:500 dilution), or anti-β-actin (1:2500, loading control) (Cell Signaling Technology, Danvers, MA, USA), or mouse monoclonal anti-apolipoprotein E (ApoE; 1:1000 dilution), anti-paraoxonase 1 (PON1; 1:1000 dilution) (Abcam, Cambridge, UK), or anti-ICAM-1 (1:1000 dilution) (Santa Cruz Biotechnology, Dallas, TX, USA) antibodies (18 h, 4 °C). After incubation, the membranes were washed and incubated with secondary antibody conjugates with horseradish peroxidase goat anti-rabbit or anti-mouse antibodies (Thermo Fisher Scientific, Waltham, MA, USA) for 1 h. The membranes were washed again, and the proteins were detected by enhanced chemiluminescence and quantified by densitometry.

### 2.7. Lipid Determination Assays

Total, ester, and free cholesterol were determined in isolated HDL from MPH and MSPH women by an AmplexRed cholesterol assay kit (Thermo Fisher, Waltham, MA, USA), as described [13]. Triglycerides were determined in isolated HDL by a serum triglyceride determination kit (Sigma-Aldrich, St. Louis, MI, USA), according to the manufacturer’s instructions. The results were expressed in μM, according to calibration curves.

### 2.8. Intracellular Reactive Oxygen Species (ROS) Determination

The antioxidant capacity of maternal HDL from MPH and MSPH women was determined by measuring intracellular ROS levels in HUVECs, as described [13]. Briefly, confluent cells grown on 96-well culture plates (seeded at 25,000 cells/well) were incubated with maternal HDL (50 μg/mL; 1 h, 37 °C), in the absence or presence of copper sulphate (CuSO_4_;10 μM, 3 h), used as a positive control. After incubation, cells were washed and incubated with the fluorescent dye CM-H_2_DCFDA (10 μM, Sigma-Aldrich, USA) for 30 min (37 °C). Fluorescence (λ_exc_/λ_em_: 485/535 nm) was determined in a Synergy H1 microplate reader, and cells were lysed with KOH. Intracellular ROS levels were expressed as relative fluorescence units (RFU) per μg of protein.

### 2.9. PON1 Enzymatic Activity

PON1 activity was determined in maternal total serum from MPH and MSPH women using the EnzChek^®^ Paraoxonase Assay Kit (Thermo Fisher, Waltham, MA, USA), according to the manufacturer’s instructions as described [22]. The results were expressed as enzymatic units (U) per μL, according to calibration curves.

### 2.10. Alpha-Tocopherol Levels

α-tocopherol levels were determined in maternal total serum from MPH and MSPH women by high performance liquid chromatography (HPLC), at the Barnafi Krause laboratory, Chile.

### 2.11. Cholesterol Efflux Capacity

The cholesterol efflux capacity of isolated maternal HDL from MPH and MSPH women was determined in HUVECs, as described [10]. Briefly, cells were seeded on 24-well culture plates (50,000 cells/well) and incubated with [3H]-cholesterol (0.5 μCi/mL) for 24 h. After incubation, cells were washed with PBS supplemented with BSA-FFA (bovine serum albumin-free fatty acid, 2 mg/mL), and incubated with maternal HDL (50 μg/mL; 6 h, 37 °C). Subsequently, the culture medium was recovered, and the cells were lysed with KOH. Cholesterol efflux was expressed as the percentage (%) of radioactivity determined in supernatants relative to the total signal (medium plus cell lysate), corrected by the protein concentration estimated in cell lysates.

### 2.12. Endothelial Activation Assay

Anti-inflammatory activity or the capacity of maternal HDL from MPH and MSPH women to inhibit endothelial activation was determined in HMEC-1, as described [19]. Cells were seeded on 6-well culture plates (350,000 cells/well), and after reaching confluence, cells were co-incubated with lipopolysaccharide (LPS; 1 μg/mL) and maternal HDL (50 μg/mL) for 18 h (37 °C), in minimum medium. Then, cells were lysed in RIPA buffer, and endothelial activation was evaluated by measuring the protein expression of ICAM-1 by Western blot.

### 2.13. Nitric Oxide Determination

Vasodilatory activity or the capacity of maternal HDL from MPH and MSPH women to stimulate nitric oxide (NO) production was assessed by measuring intracellular NO and by evaluating nitric oxide synthase (eNOS) expression [8].

Intracellular NO was determined in confluent HMEC-1 grown on 96-well culture plates (seeded at 25,000 cells/well), incubated with maternal HDL (50 μg/mL) for 18 h (37 °C), in minimum medium. Then, L-Arginine (L-Arg, 100 μM) and L-Arg plus N(ω)-nitro-L-arginine methyl ester (L-NAME; NOS inhibitor; 100 μM) were added, along with the fluorescent dye DAF-FM diacetate (5 μM; Invitrogen, Waltham, MA, USA) for 1 h (37 °C). Fluorescence (λ_exc_/λ_em_: 490/525 nm) was determined in a Synergy H1 microplate reader, and cells were lysed with KOH. Intracellular NO was expressed as RFU per μg of protein, as described [19].

eNOS expression was determined in confluent HMEC-1 seeded on 6-well culture plates (350,000 cells/well) and incubated with HDL (50 μg/mL) for 18 h (37 °C), in minimum medium. Cells were lysed in RIPA buffer, and the protein expression of total eNOS and eNOS phosphorylated at Serine^1177^ (p-Ser-eNOS) was evaluated by Western blot [8].

### 2.14. Inflammation Marker Determination

Interleukin-1β (IL-1β), IL-6, IL-8, IL-10, IL-12p70, and tumor necrosis factor (TNF) levels were determined in maternal total serum from MPH and MSPH women by flow cytometry using the Cytometric Bead Array (CBA) Human Inflammatory Cytokine Kit (BD, Biosciences, Franklin Lakes, NJ, USA), as described [23]. The results were expressed as the mean fluorescence intensity (MFI).

### 2.15. Apolipoprotein Determination

Apolipoprotein AI (ApoAI), ApoAII, ApoB, ApoCII, ApoCIII, and ApoE levels were determined in maternal total serum from MPH and MSPH women by Luminex using the MILLIPLEX Human Apolipoprotein Magnetic Bead Panel—Cardiovascular Disease Multiplex Assay (APOMAG-62K; Merck Millipore, Billerica, MA, USA), as described [24]. The results were expressed as g/L, according to calibration curves.

### 2.16. Endothelial Dysfunction Marker Determination

Soluble ICAM-1 and VCAM-1 (sICAM-1 and sVCAM-1, respectively) levels were determined in maternal total serum from MPH and MSPH women by ELISA kits (Human ICAM-1/CD54 Allele-specific Quantikine ELISA Kit and Human VCAM-1/CD106 Quantikine ELISA Kit, respectively; R&D Systems, Minneapolis, MN, USA), as described [25]. The results were expressed as ng/mL, according to calibration curves.

### 2.17. Determination of Total Antioxidant Capacity in Plasma

Antioxidant status was assessed by the ferric reducing ability of plasma (FRAP), as described [26]. Briefly, 750 μL of FRAP solution (300 mmol/L of acetate buffer, 10 mmol/L of 2,4,6-tri(2-pyridyl)-1,3,5-triazine solution, and 20 mmol/L of FeCl_3_·6H_2_O in a 10:1:1 ratio) was mixed with 75 μL H_2_O and 25 μL of serum and then incubated at 25 °C for 30 min. The absorbance was determined at 593 nm. A standard curve of FeSO_4_ was prepared for the quantitative determination of FeSO_4_ as Fe^2+^ equivalents produced in the samples.

### 2.18. Cardiovascular Risk Markers

#### 2.18.1. ApoB/ApoAI Ratio

ApoB/ApoAI ratio [27,28] was calculated from the values of ApoB and ApoAI obtained in the maternal total serum from MPH and MSPH women, as described above.

#### 2.18.2. Atherogenic Index of Plasma (AIP)

Atherogenic index of plasma (AIP) was calculated as the logarithmically transformed ratio of the molar concentrations of TG and HDL-C (log (TG/HDL-C)) [29,30], determined in maternal total serum from MPH and MSPH women, as described above. AIP values less than 0.11 were considered as low cardiovascular risk, between 0.11 and 0.21 as intermediate risk, and over 0.21 as high cardiovascular risk.

### 2.19. Statistical Analysis

The values for maternal and neonatal characteristics are presented as the mean ± S.D., as described [13]. For in vitro assays, the values are presented as the mean ± S.E.M., where *n* indicates the number of samples (serum or isolated HDL) used per group. The normality of the data was determined with a Kolmogorov–Smirnov’s test. Comparisons between groups were determined by a Student’s t-test (serum determinations) and Mann–Whitney test (assays with isolated HDLs) for parametric and nonparametric data, respectively. *p* < 0.05 was considered statistically significant. GraphPad Prism 7.0 statistical software (GraphPad Software Inc., San Diego, CA, USA) was used for data analysis.

## 3. Results

### 3.1. Clinical Characteristics of the Participants

In total, 57 pregnant women without diagnosed pathologies were considered in this study. Pregnant women were classified into the MPH (TC < 280 mg/dL; *n* = 34) or MSPH (TC ≥ 280 mg/dL; *n* = 23) groups, as described in the previous section. Regarding the maternal and neonatal clinical variables analyzed (Table 1), the differences between the two groups were only observed in maternal lipids at delivery. Specifically, TC and LDL levels were significatively higher (37% and 64.7%, respectively) in women from the MSPH group than the MPH group.

### 3.2. Composition of Maternal HDL from MPH and MSPH Pregnancies

**Figure 1:**
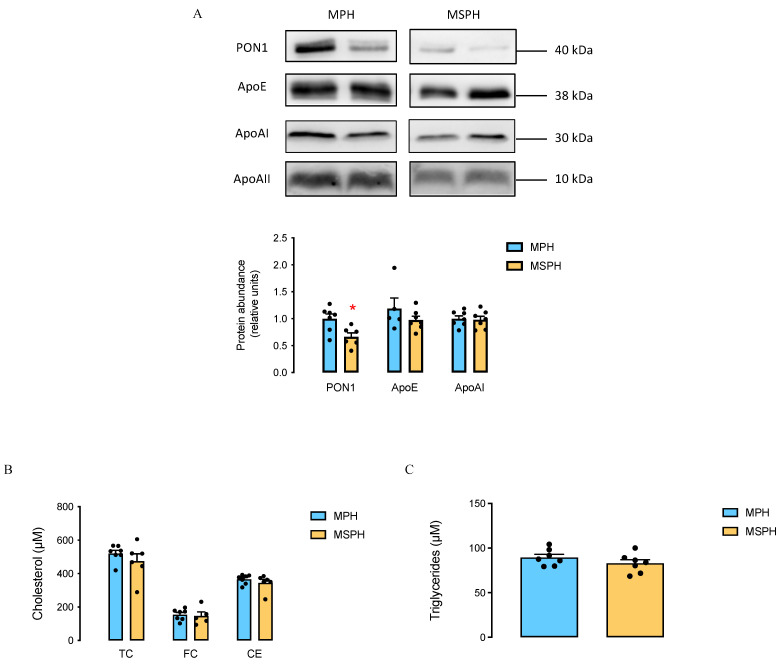
Composition of maternal HDL from MPH and MSPH women. (**A**) Representative Western blot showing the protein abundance in HDL isolated from MPH and MSPH maternal serum. An equal quantity of protein (30 μg) was loaded for each sample. Paraoxonase 1 (PON1), apolipoprotein E (ApoE), and apolipoprotein AI (ApoAI) were determined; apolipoprotein AII (ApoAII) was used as a loading control (*n* = 4 per group). Western blot quantification for specific proteins relative to ApoAII is shown in the bar graph (MPH, light blue bars, and MSPH, yellow bars). (**B**) Levels of cholesterol: total (TC), free (FC), and ester (CE) in HDL isolated from MPH (light blue bars) and MSPH (yellow bars) maternal serum. (**C**) Triglyceride levels determined in HDL isolated from MPH (light blue bar) and MSPH (yellow bar) maternal serum. *n* = 7 per group. Values are the mean ± S.E.M. * *p* < 0.05 vs. corresponding values in the MPH group.

HDL was isolated from MPH and MSPH serum (*n* = 7 per group), and equal protein mass was separated by electrophoresis. The Western blot quantification of ApoAI, ApoE, and PON1 relative to ApoAII showed a lower protein abundance of PON1 (42.9%) in isolated HDL from MSPH women compared to HDL from MPH women (Figure 1A). No significant differences were observed in the cholesterol or triglyceride content of isolated HDL from MPH and MSPH women (Figure 1B,C).

### 3.3. Biological Activities of Maternal HDL from MPH and MSPH Pregnancies

**Figure 2:**
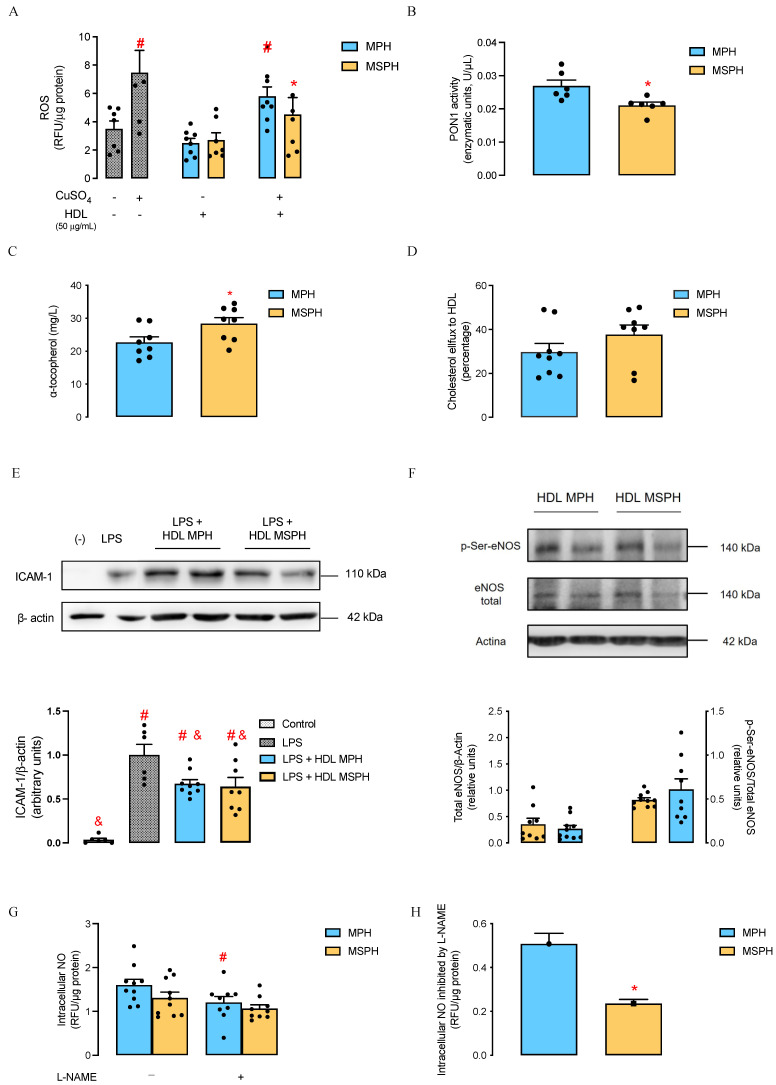
Biological activities of maternal HDL from MPH and MSPH women. (**A**) Antioxidant capacity of HDL from MPH and MSPH women. The levels of reactive oxygen species (ROS) were determined in control human vein endothelial cells (HUVECs; dotted bar) or HUVECs incubated with CuSO_4_ (dotted bar), or with HDL isolated from MPH (light blue bars) or MSPH (yellow bars) maternal serum, in the presence or absence of CuSO_4_. (**B**) Enzymatic activity of the antioxidant enzyme paraoxonase 1 (PON1) and (**C**) α-tocopherol levels (antioxidant vitamin), determined in the total serum of MPH (light blue bars) and MSPH (yellow bars) women. (**D**) Cholesterol efflux capacity of HDL from MPH (light blue bar) and MSPH (yellow bar) women, determined in HUVECs incubated with tritiated cholesterol. (**E**) Anti-inflammatory activity of HDL from MPH and MSPH women. Representative Western blot and blot quantification showing the protein abundance of intercellular cell adhesion molecule 1 (ICAM-1), determined in control human dermal microvascular endothelial cell 1 (HMEC-1; dotted bar), HMEC-1 incubated with lipopolysaccharide (LPS; dotted bar), or HMEC-1 co-incubated with LPS and HDL isolated from MPH (light blue bar) or MSPH (yellow bar) maternal serum. (**F**–**H**) Effects of HDL from MPH and MSPH women in nitric oxide activity. (**F**) Representative Western blot and blot quantification showing the protein abundance of total and p-Ser1177-eNOS, determined in HMEC-1 incubated with HDL isolated from MPH (light blue bar) and MSPH (yellow bar) maternal serum. β-actin was used as the loading control. (**G**) Intracellular levels of nitric oxide (NO) in HMEC-1 incubated with HDL isolated from MPH (light blue bars) or MSPH (yellow bars) maternal serum, in the presence or absence of NG-Nitro-L-arginine methyl ester (L-NAME, nitric oxide synthase (NOS) inhibitor). (**H**) NOS activity estimated as the fraction of intracellular NO inhibited by L-NAME in HMEC-1 incubated with HDL from MPH (light blue bar) and MSPH (yellow bar) women. Values are the mean ± S.E.M. # *p* < 0,05 vs. basal levels of ROS, ICAM-1, or NO for (**A**,**E**,**G**), respectively; * *p* < 0.05 vs. corresponding values in the MPH group; & *p* < 0.05 vs. LPS condition (*n* = 4–10 per group).

The antioxidant capacity of maternal HDL was determined by measuring the levels of ROS in HUVECs incubated with HDL from MPH and MSPH women, in the presence or absence of CuSO_4_, used as a positive control (Figure 2A). Lower levels of ROS were observed with both HDL from MPH and MSPH pregnancies in the absence of CuSO_4_, compared to the positive control, these levels being similar to the basal condition. When cells were co-incubated with HDL and CuSO_4_, lower levels of ROS were observed with HDL from MSPH women (39.6%) compared to the cells with CuSO_4_. In addition, reduced PON1 enzymatic activity (Figure 2B) and increased levels of α-tocopherol (Figure 2C) were determined in the total serum of MSPH women (28.6% and 25.1%, respectively) compared to MPH.

Furthermore, when the cholesterol efflux capacity of maternal HDL was assessed, HDL from MSPH women tended to have a greater ability to remove cholesterol from HUVECs (*p* = 0.1975) compared to HDL MPH (Figure 2D).

The anti-inflammatory activity of maternal HDL was determined by evaluating ICAM-1 protein expression in HMEC-1 incubated with HDL from MPH and MSPH women, in the presence of LPS to induce endothelial activation (Figure 2E). An increased protein abundance of ICAM-1 was observed in cells co-incubated with LPS and HDL from both MPH and MSPH pregnancies compared to the basal expression. These levels were lower compared to HMEC-1 incubated with LPS, with no difference between the MPH and MSPH groups.

The maternal HDL capacity to stimulate nitric oxide production was evaluated by determining the protein expression of total and p-Ser1177-eNOS (Figure 2F), the intracellular levels of NO (Figure 2G,H) in HMEC-1 incubated with HDL isolated from MPH and MSPH women. No changes were observed in the expression of p-Ser1177-eNOS or total eNOS between HDL MPH and MSPH. Moreover, when cells were incubated with maternal HDL in the presence or absence of L-NAME, the inhibition was higher in cells incubated with HDL from MPH women compared to MSPH (Figure 2G). Finally, the intracellular NO levels inhibited by L-NAME (i.e., NOS activity) were 53.7% lower when cells were incubated with HDL from MSPH women compared to MPH (Figure 2H).

### 3.4. Cytokine Levels in Maternal Serum

IL-1β, IL-6, IL-8, IL-10, IL-12p70, and TNF levels were determined in maternal serum from MPH and MSPH women (*n* = 15 per group). Both IL-10 and IL-12p70 levels were significatively higher (15% and 16%, respectively) in MSPH serum compared to MPH serum (Table 2).

### 3.5. Apolipoprotein Levels in Maternal Serum

ApoAI, ApoB, ApoAII, ApoE, ApoCII, and ApoCIII levels were determined in maternal serum from MPH and MSPH women (*n* = 10 per group). The levels of ApoB, ApoCII, and ApoCIII were significantly higher (41.9%, 53.3%, and 22.4%, respectively) in the MSPH maternal serum compared to MPH (Table 3).

### 3.6. Markers of Endothelial Dysfunction, Antioxidant Capacity, and Cardiovascular Risk in Maternal Serum

**Figure 3:**
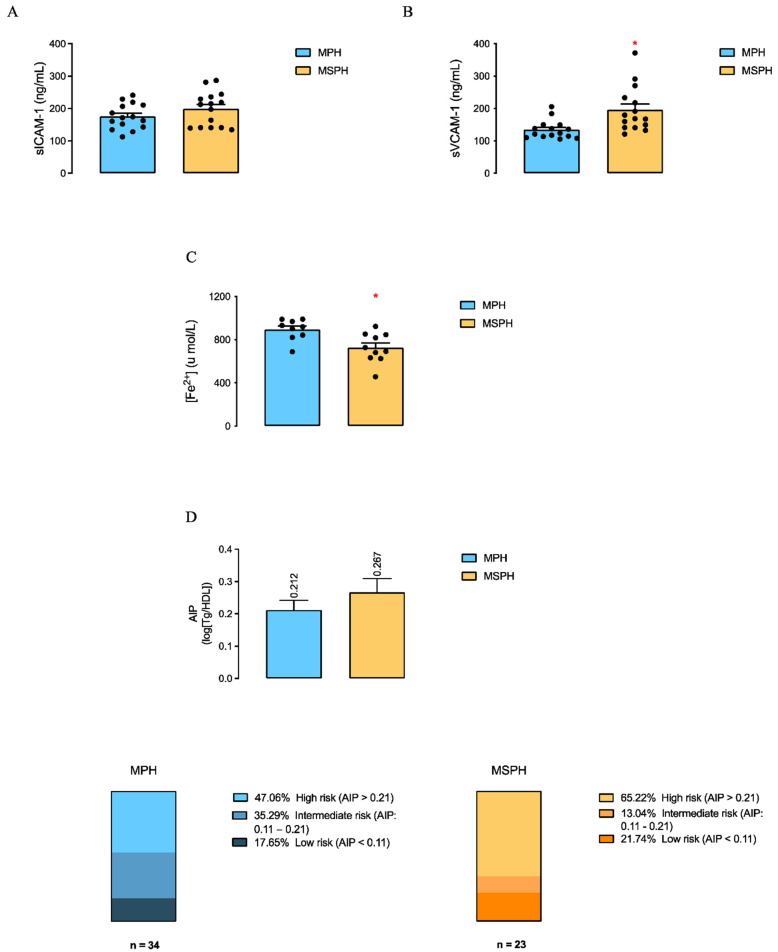
Markers of endothelial dysfunction, total antioxidant capacity, and cardiovascular risk of MPH and MSPH women. The levels of (**A**) soluble intercellular cell adhesion molecule 1 (sICAM-1) and (**B**) soluble vascular cell adhesion molecule 1 (sVCAM-1) were determined in the total serum of MPH (light blue bars) and MSPH (yellow bars) women (*n* = 15 per group). (**C**) Serum total antioxidant capacity was determined in MPH (light blue bars) and MSPH (yellow bars) women (*n* = 10 per group). (**D**) Cardiovascular risk was determined in MPH and MSPH women by the atherogenic index of plasma (AIP) expressed as the mean AIP (light blue and yellow bars) and as the percentage of distribution between high, intermediate, and low risk (MPH, *n* = 34, and MSPH, *n* = 23). Values are the mean ± S.E.M. * *p* < 0.05 vs. corresponding values in the MPH group.

sICAM-1 and sVCAM-1 levels were measured in maternal serum from MPH and MSPH women (*n* = 15 per group; Figure 3). The results showed that sVCAM-1 was 26% higher in the maternal serum from MSPH women compared to MPH women (Figure 3B), without differences in sICAM-1 levels (Figure 3A).

The total antioxidant capacity of maternal serum was determined in MPH and MSPH samples. Figure 3C shows that the serum of MSPH women presented a 19% lower antioxidant capacity compared to MPH women.

The cardiovascular risk of MPH and MSPH women was evaluated by the ApoB/ApoAI ratio and AIP. The ApoB/ApoAI ratio was 69.9% higher in MSPH than MPH pregnant women (Table 3). No differences were determined in the mean AIP between MPH (*n* = 34) and MSPH (*n* = 23) women (Figure 3D), but the percentage of women with high cardiovascular risk (AIP > 0.21) tended to be higher in the MSPH group compared to MPH (65.2% vs. 47.1%, respectively) (Figure 3D).

## 4. Discussion

In this report, we show for the first time the structural and functional characteristic of HDL from MSPH women at term of pregnancy. In summary, HDL isolated from MSPH women had a lower protein abundance and a reduced activity of the antioxidant enzyme PON1; however, an increased antioxidant capacity was observed compared to HDL from MPH women, along with higher serum levels of the antioxidant vitamin α-tocopherol. Moreover, HDL from MSPH women had a lower capacity to induce the formation of NO in endothelial cells compared to HDL from MPH. Additionally, we observed augmented levels of ApoB, ApoCII, and ApoCIII, increased levels of IL-10 and IL-12p70, and an increased ApoB/ApoAI ratio (i.e., higher cardiovascular risk) in maternal serum from MSPH compared to MPH women.

We recruited 57 pregnant women, 34 of them being classified in the MPH group and 23 in the MSPH group according to their TC levels at term of pregnancy. Table 1 indicates that no differences were observed between the groups when analyzing the different clinical parameters such as glycemia, blood pressure, and body weight, suggesting that the reported results in this manuscript would not be related to pregnancy-related diseases such as obesity, GDM, or preeclampsia and thus must be related to the main differences among both groups of women: the lipid levels.

When the maternal lipid profile was analyzed, higher levels of TC and LDL cholesterol were determined in the MSPH group compared to the MPH, a result consistent with data previously reported by us [2,6,7,10,13,19] and other groups of study [1,5,11,31,32]. It is important to highlight that the women who participated in this study did not have a previously reported clinical history of hypercholesterolemia. Therefore, the increase in cholesterol levels, both in MPH and MSPH women, would not be related to a previous condition of hypercholesterolemia. We previously published that in MSPH women, the elevated levels of LDL could be related to increased maternal levels of proprotein convertase subtilisin/kexin type 9 (PCSK9), as previously reported [13]. Other mechanisms leading to the genesis of MSPH are unknown. Interestingly, it has been reported that in animal models and humans, the exposition to endocrine disruptors associates with increased levels of cholesterol, which also occurs in pregnancy [33,34,35,36]. These data suggest that the quantification of exposition to endocrine disruptors in MSPH women could contribute to understand other possible mechanisms leading to this metabolic maternal alteration, which need to be determined.

Despite that the mechanisms leading to MSPH are currently not fully understood, the study of the effects of this maternal metabolic alteration needs also be determined at different levels. In a previous paper of our group, it was reported that maternal LDLs from MSPH women were pro-atherogenic particles that contributed to a pro-oxidative profile of these women [13]. Although the levels of HDL cholesterol were similar in MPH and MSPH women, in this work, we aimed to know whether the HDL of these MSPH women exhibits changes in their composition and anti-atherogenic functions compared to MPH.

First, when the protein composition of maternal HDL was evaluated, a lower protein abundance of the antioxidant enzyme PON1 was observed in HDL from MSPH women compared to MPH, which suggests that these lipoproteins could have a lower antioxidant function. Other studies have also reported a lower abundance of PON1 in the HDL of pregnant women compared to non-pregnant women [18] and in the HDL of pregnancies with GDM compared to normal pregnancies [22]; therefore, this result constitutes the first evidence to suggest that the MSPH condition could also be a factor that affects the presence of PON1 in HDL. On the other hand, although we did not observe differences in the cholesterol and triglyceride content of HDL from MPH and MSPH women, it might be relevant to further study the abundance of the other lipids of these lipoproteins, for example, their phospholipid and sphingolipid content, since these lipid components are also involved in several biological activities of HDL [21], and it was recently reported that the lipidomic profiles of maternal blood were different between MPH and MSPH [32].

Moreover, although HDL functions have been widely studied in adults [15,20], little is known during pregnancy, this work being the first to study changes in the function and effects of maternal HDL on endothelial cells under conditions of hypercholesterolemia during pregnancy. First, we evaluated the antioxidant capacity of HDL in endothelial cells by determining ROS levels in oxidative conditions. Our results suggest that HDLs from MSPH women have a greater antioxidant capacity compared to MPH, as lower ROS levels were determined in cells incubated with those lipoproteins. Considering that we determined that HDL MSPH has a lower protein abundance and a lower activity of the antioxidant enzyme PON1, the greater antioxidant capacity could be a mechanism to counteract the pro-oxidative environment of MSPH [13] and could be due to the presence of other antioxidant systems relevant to the function of these lipoproteins. One possibility is that the increased α-tocopherol levels in MSPH women could contribute to the antioxidant capacity of their HDL, as it has been reported that HDL can play a significant role in the transport of vitamin E [37,38,39]. Also, enzymes such as glutathione selenoperoxidase (GSPx), platelet activating factor (PAF-AH), and lecithin–cholesterol acyltransferase (LCAT) may contribute to the greater antioxidant capacity of HDL MSPH, as it has been reported in adults [20,21], which requires to be determined. Despite that, the evaluation of serum total antioxidant capacity was decreased in MSPH women, suggesting that the increased antioxidant capacity of HDL could be counteracted in these women, which could be related to previously reported increased levels of oxidized LDL [13].

Additionally, during pregnancy, a greater efflux capacity has been reported in HDL from pregnant women compared to non-pregnant women [18], while HDL from women with GDM presented a lower capacity compared to lipoproteins from normal pregnancies [22]. In our study, we did not observe differences in the cholesterol efflux capacity of HDL from MSPH compared to MPH women, a result that could be in line with the fact that we did not observed changes in the abundance of ApoAI and ApoE in HDL from MSPH, as those apolipoproteins play a key role in the cholesterol efflux capacity [20,21]. Moreover, considering that MSPH women are exposed to higher levels of cholesterol during pregnancy, it is possible to hypothesize that the unaltered cholesterol efflux capacity of HDL might not be sufficient to remove the excess of cholesterol; in fact, a higher cholesterol content has been reported in placental cells from MSPH women compared to MPH [10], suggesting that the efflux mediated by HDL is not enough.

Finally, although we have not observed differences in the anti-inflammatory activity of HDL from MPH and MSPH women, here we reported that maternal HDL from MSPH pregnancies has a reduced nitric oxide modulatory activity compared to HDL from MPH. Specifically, in endothelial cells, we observed that HDL from MSPH had a lower capacity to induce the formation of NO by eNOS, by a mechanism that would be independent of its classical activation in Ser^1177^, as we did not observed changes in the protein expression of total and p-Ser1177-eNOS. Interestingly, it has been reported that HDL from patients with coronary disease has a lower capacity to stimulate the endothelial production of NO as a result of a lower PON1 activity, in a mechanism that involves the activation of lectin-type oxidized LDL receptor 1 (LOX-1) and protein kinase C-βII (PKCβII) [40]. Thus, we proposed that our results could be consistent with the activation of this pathway by HDL from MSPH women, which presents a lower PON1 activity compared to HDL from MPH pregnant women.

Given our previous and current findings that MSPH alters both the composition and function of maternal LDL [13] and HDL, we then aimed to know whether these changes in maternal lipoprotein metabolism could be accompanied by changes at the serum level, such as endothelial dysfunction, total antioxidant capacity, and inflammatory markers, i.e., maternal vascular dysfunction. As shown, higher levels of ApoB, ApoCII, and ApoCIII were described in MSPH serum compared to MPH. The increase in ApoB levels is consistent with what has been determined in pregnant women compared to non-pregnant women [41,42] and with what was reported by Marseille-Tremblay [31] in MPH and MSPH women. We propose that these changes could have consequences in the metabolism of maternal HDL and in the distribution of its subspecies, since an increase in the concentration of ApoB, ApoCII, and ApoCIII has been reported to result in elevated levels of small HDL and lower levels of large HDL [43,44]. However, both in adults [45] and pregnant women [18], it has been shown that ApoCII and ApoCIII are found preferentially in HDL2 or larger HDL. Thus, even though the distribution of these proteins in HDL is not entirely clear, there is evidence that they modify the distribution of subpopulations of these lipoproteins and, consequently, we propose that the subpopulations of HDL could be different in MSPH women compared to MPH, which requires to be confirmed.

To the best of our knowledge, this is the first report of the existence of a reduced total antioxidant capacity and changes in markers of vascular dysfunction in MSPH women compared to MPH. Specifically, here, we reported a lower antioxidant capacity in serum from MSPH women, a result that shows by a different approach that in MSPH the redox status is impaired in MSPH women, which could be related among other factors to increased PCSK9 levels, as previously described [13,26,46]. In terms of endothelial dysfunction, higher levels of sVCAM-1 were determined in the serum of MSPH women compared to MPH, which is consistent with the increased levels of this endothelial marker reported in non-pregnant adults with hypercholesterolemia [47]. Furthermore, higher levels of sVCAM-1 and sICAM-1 have been reported in women with preeclampsia [48,49,50,51,52] and intrauterine growth restriction [53] compared to normal pregnancies, studies in which it has been proposed that abnormal levels of endothelial dysfunction markers could have a predictive value. Similarly, we propose that the levels of these adhesion molecules may have a predictive value associated with maternal cholesterol levels during pregnancy. Also, here, we observed higher levels of both the pro-inflammatory cytokine IL-12p70 and the anti-inflammatory cytokine IL-10 in the serum of MSPH women compared to MPH. In accordance, in women with preeclampsia, it has been proposed that higher levels of IL-10 could be associated with a compensatory mechanism due to an increase in pro-inflammatory cytokines [52].

Finally, in the maternal serum, we analyzed two cardiovascular risk markers, the ApoB/ApoAI ratio and AIP, which have been proposed as better predictors of CVD compared to lipid levels [54,55,56]. In MSPH women, in line with a higher serum ApoB concentration, without changes in ApoAI shown here and previously [31], we observed a higher ApoB/ApoAI ratio compared to MPH, as it has been reported in women with preeclampsia compared to normal pregnancies [57] and in pregnant women compared to non-pregnant women [58]. Thus, this result suggests that MSPH women have a higher cardiovascular risk during pregnancy compared to MPH women. Moreover, although we did not observe differences in the average AIP between the MSPH and MPH groups, when analyzing the risk distribution, it is possible to suggest that a higher percentage of MSPH women could present a high cardiovascular risk compared to MPH. Therefore, all the exposed results suggest the possibility of an increased cardiovascular risk in MSPH, which needs to be evaluated also at the postpartum period to establish if the changes are circumscriptive only to pregnancy or are permanent before.

The main limitation of this study relates to the origin of the biological samples that corresponds to women of one region of a particular country. Therefore, the data need to be validated in a multicentric study.

## 5. Conclusions

This study shows for the first time that the supraphysiological increase in maternal cholesterol levels is related with markers of endothelial dysfunction and atherogenic risk factors in pregnant women at the third trimester of pregnancy, along with changes in both the composition and anti-atherogenic functions of maternal HDL in endothelial cells. Thus, this work contributes to broaden the knowledge regarding the effects of high cholesterol levels during pregnancy on maternal lipoprotein metabolism, which eventually could impair maternal vascular health during pregnancy or after, which needs to be determined in future assays. Based on that hypothesis, we propose that the monitoring of lipid levels during pregnancy could contribute to the understanding of their effect on offspring and also the mother.

## Figures and Tables

**Table 1:** Clinical characteristics of pregnant women and their newborns. Women with maternal physiological (MPH, TC ≤ 280 mg/dL) or supraphysiological hypercholesterolemia (MSPH, TC > 280 mg/dL) at delivery were included (see Section 2). Weight, body mass index (BMI), and blood pressure were determined at trimester (T) 1, 2, and 3. Lipid profiles were determined at delivery. OGTT, oral glucose tolerance test; HDL, high-density lipoprotein; LDL, low-density lipoprotein; VLDL, very-low-density lipoprotein. * *p* < 0.05 vs. corresponding values in the MPH group. Data are presented as the mean ± S.D. (range).

Group	MPH (*n* = 34)	MSPH (*n* = 23)
**Maternal variables**		
Weeks of gestation	38.9 ± 0.9 (37–41)	39.4 ± 0.8 (38–41)
Age (years)	31.4 ± 4.8 (22–40)	31.1 ± 5.8 (24–48)
Height (cm)	160.8 ± 6.5 (150–173)	160 ± 8.1 (148–180)
Weight (kg)		
T1	61.7 ± 8.1 (48–81)	59.6 ± 8.7 (47–82)
T2	67.7 ± 7.3 (54–87)	65.5 ± 8 (50–82)
T3	71.9 ± 7.3 (61–86)	70.1 ± 7.1 (55–85)
BMI (kg/m^2^)		
T1	23.8 ± 2.5 (19.5–29.7)	23.2 ± 2.4 (18.6–29)
T2	26.2 ± 2 (23.1–30.9)	25.6 ± 2.4 (19.8–30.1)
T3	27.8 ± 2.1 (23.8–33)	27.3 ± 2.3 (21.8–30.9)
Weight gain (kg)	10.2 ± 3.1 (2.3–16)	10.4 ± 3.6 (3–16)
Systolic arterial pressure (mm Hg)		
T1	109.3 ± 9.7 (90–120)	110.1 ± 9.5 (100–130)
T2	109.4 ± 9.1 (90–125)	106.8 ± 9.7 (90–130)
T3	110.9 ± 8.4 (100–130)	107 ± 9.3 (96–130)
Diastolic arterial pressure (mm Hg)		
T1	67 ± 7.2 (50–80)	68.8 ± 8 (60–80)
T2	67.2 ± 6.1 (60–80)	64.1 ± 8.5 (40–80)
T3	69.5 ± 7.4 (53–80)	67.9 ± 8.8 (55–90)
OGTT (mg/dL)		
Basal glycaemia	77.1 ± 8.5 (61–93)	76.6 ± 6.4 (68–90)
Glycaemia 2 h after glucose	103.6 ± 19.3 (71–135)	101.5 ± 18.1 (65–129)
Parity		
Primiparous	11 (32.4%)	8 (34.8%)
1	18 (52.9%)	11 (47.8%)
≥2	5 (14.7%)	4 (17.4%)
Lipid levels at delivery (mg/dL)		
Total cholesterol	232.7 ± 34.5 (168–280)	318.9 ± 28.1 * (285–402)
HDL	62.2 ± 12.8 (35–106)	63.2 ± 15.9 (33–94)
LDL	122.8 ± 31.5 (61–169)	202.2 ± 26.3 * (157–273)
VLDL	48 ± 12.4 (25–77)	53.6 ± 13.1 (27–79)
Triglycerides	239.8 ± 62.1 (124–385)	267.4 ± 65.4 (133–393)
**Newborn variables**		
Sex (female/male)	16/18	10/13
Birth weight (g)	3375 ± 363.5 (2705–4230)	3545 ± 387.9 (2750–4375)
Height (cm)	49.2 ± 1.7 (46–52)	49.8 ± 1.2 (48–52)
Ponderal index (g/cm^3^ × 100)	2.8 ± 0.2 (2.4–3.3)	2.9 ± 0.2 (2.5–3.2)

**Table 2:** Cytokine levels (MFI) in maternal serum from MPH and MSPH women. Levels of interleukin-1β (IL-1β), IL-6, IL-8, IL-10, IL-12p70, and tumor necrosis factor (TNF) were determined in maternal serum from MPH and MSPH pregnant women by flow cytometry (see Methods). * *p* < 0.05 vs. corresponding values in the MPH group. Results are expressed as the mean fluorescent intensity (MFI) ± S.D.

Cytokine	MPH (*n =* 15)	MSPH (*n =* 15)
IL-1β	3.71 ± 0.67	3.66 ± 1.72
IL-6	3.54 ± 1.07	3.99 ± 1.33
IL-8	9.7 ± 5.24	10.1 ± 8.13
IL-10	2.54 ± 0.46	2.92 ± 0.39 *
IL-12p70	1.5 ± 0.32	1.74 ± 0.27 *
TNF	1.84 ± 0.53	1.81 ± 0.37

**Table 3:** Apolipoprotein levels (g/L) in maternal serum from MPH and MSPH women. Levels of apolipoprotein AI (ApoAI), ApoB, ApoAII, ApoE, ApoCII, and ApoCIII were determined in maternal serum from MPH and MSPH pregnant women (see Methods), expressed in g/L. ApoB/ApoAI ratio was determined as a cardiovascular risk marker. * *p* < 0.05 vs. corresponding values in the MPH group. Values are the mean ± S.D.

Apolipoprotein	MPH (*n* = 10)	MSPH (*n* = 10)
ApoAI	1.15 ± 0.38	1.05 ± 0.32
ApoB	1.67 ± 0.45	2.37 ± 0.58 *
ApoAII	0.24 ± 0.04	0.27 ± 0.048
ApoE	0.05 ± 0.013	0.06 ± 0.014
ApoCII	0.15 ± 0.047	0.23 ± 0.062 *
ApoCIII	0.49 ± 0.08	0.6 ± 0.1 *
ApoB/ApoAI	1.43 ± 0.52	2.43 ± 0.93 *

## Data Availability

The data presented in this study are available on request from the corresponding author. The data are not publicly available to maintain patients’ privacy information.
